# A pancreaticobiliary maljunction captured on video by peroral cholangioscopy

**DOI:** 10.1055/a-2719-3121

**Published:** 2025-11-04

**Authors:** Ye Zhu, Juan Liu, Rui-Hua Shi

**Affiliations:** 1 162752 Department of Gastroenterology, Zhongda Hospital, School of Medicine, Southeast University, Nanjing, China

Pancreaticobiliary maljunction (PBM) is a congenital anomaly defined as a junction of the pancreatic and bile ducts located outside the duodenal wall, usually forming a markedly long common channel 1 . Anatomically, PBM can be classified into B-P type and P-B type, in which the bile duct joins the pancreatic duct (B-P) and the pancreatic duct joins the bile duct (P-B), respectively; cases that fit neither are called complex type 2 . Here, we present a patient with B-P type PBM whose anomalous confluence was directly visualized by peroral cholangioscopy.

A 28-year-old female presented to a local hospital 2 months ago with right upper quadrant pain. MRCP ( Fig. 1 ) revealed significant dilation of the common bile duct (CBD) and pancreatic duct, with enlargement of the pancreatic head. The patient underwent ERCP at a local hospital, during which cholangiography revealed stenosis of the distal CBD. Over the past 2 months, the patient continued to experience intermittent pain. Notably, the patient had a history of acute pancreatitis 2 years ago.

**Fig. 1:**
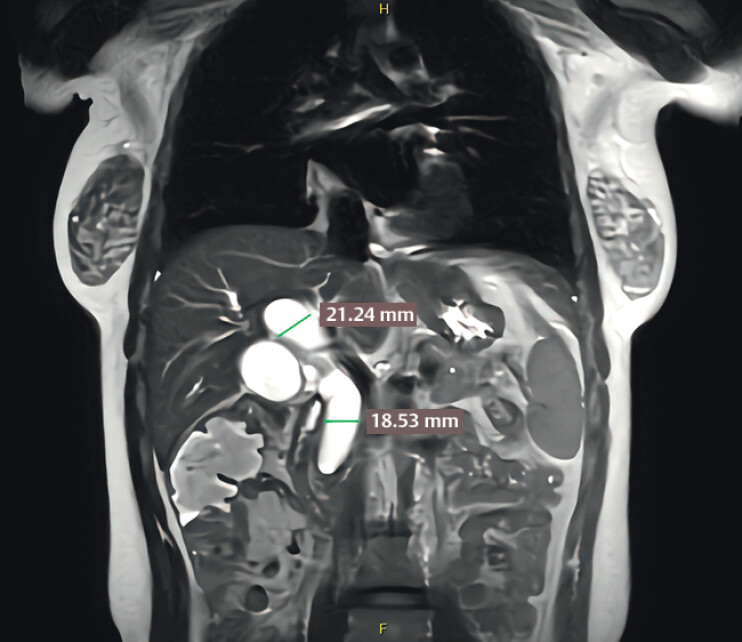
MRCP revealed significant dilation of the common bile duct.

Upon admission, the patient underwent a diagnostic ERCP and peroral cholangioscopy ( Video 1 ). During cannulation, the guidewire was repeatedly inserted into the pancreatic duct under X-ray. We then advanced the peroral cholangioscope for inspection. Careful examination identified the biliary orifice on the lateral wall of the pancreatic duct ( Fig. 2 ), with visible bile flow into the pancreatic duct. The guidewire was then advanced into the CBD, followed by the cholangioscope. Dilation of the upper segment of the CBD was observed, with rough mucosa at the end of the CBD. The length of the common channel was measured to be 3.5 cm using cholangioscopy, which was further confirmed by fluoroscopy ( Fig. 3 ). A biliary stent and a pancreatic stent were subsequently placed. Postoperatively, the patient experienced a mild elevation of serum amylase levels, which improved soon. Elective surgery is recommended, but the patient has remained asymptomatic postoperatively and is still undecided to date.

Process of peroral cholangioscopy.Video 1

**Fig. 2:**
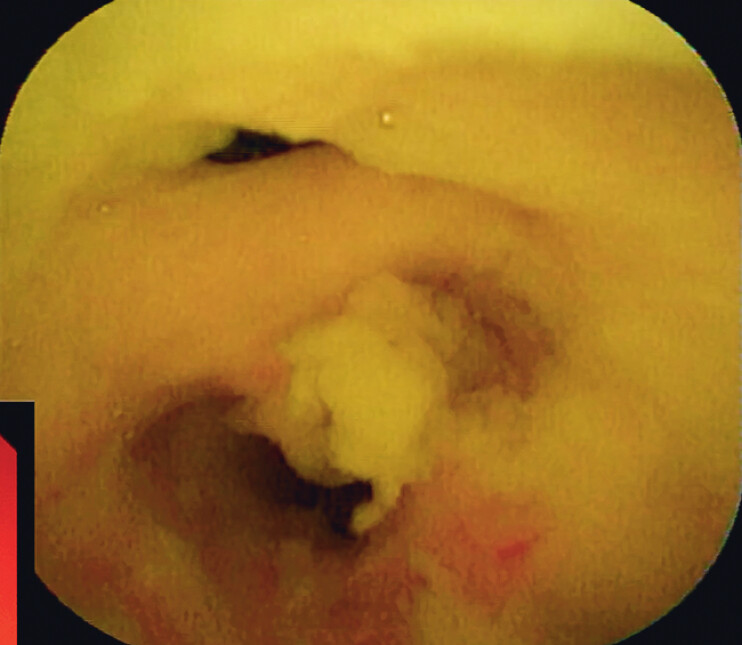
Biliary orifice on the lateral wall of the pancreatic duct (the upper one is the opening of the CBD, and the lower channel is the pancreatic duct).

**Fig. 3:**
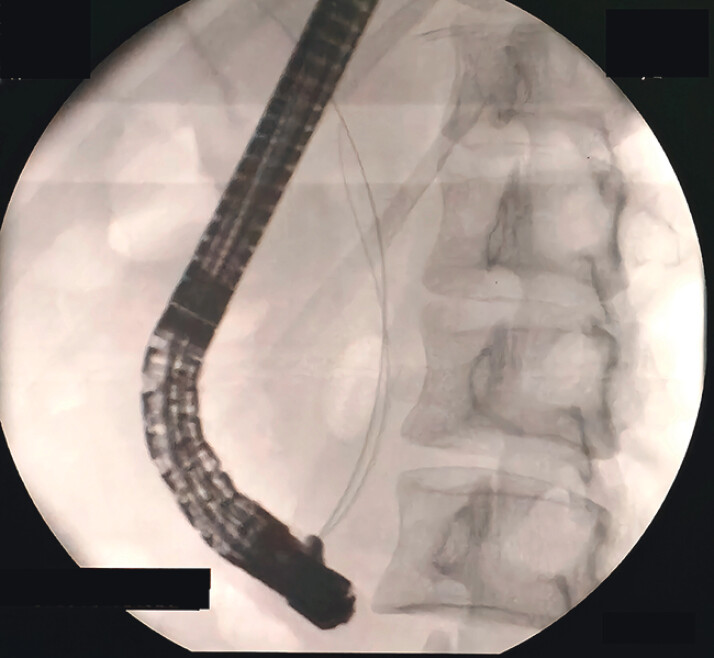
Fluoroscopy shows two guidewires (one in the pancreatic duct and the other in common bile duct) running in parallel for approximately 3.5 cm.

Endoscopy_UCTN_Code_CCL_1AZ_2AL

## Notes

Endoscopy E-Videos https://eref.thieme.de/e-videos
*E-Videos* is an open access online section of the journal *Endoscopy* , reporting on interesting cases and new techniques in gastroenterological endoscopy. All papers include a high-quality video and are published with a Creative Commons CC-BY license. Endoscopy E-Videos qualify for HINARI discounts and waivers and eligibility is automatically checked during the submission process. We grant 100% waivers to articles whose corresponding authors are based in Group A countries and 50% waivers to those who are based in Group B countries as classified by Research4Life (see: https://www.research4life.org/access/eligibility/ ). This section has its own submission website at https://mc.manuscriptcentral.com/e-videos .
